# Repurposing hydrochlorothiazide (HCTZ) for colorectal cancer: a retrospective and single center study

**DOI:** 10.3389/fphar.2025.1449062

**Published:** 2025-02-28

**Authors:** Dominic Chi-Chung Foo, Jiaxi Li, Zheng Huang, Siming Sui, Ryan Wai-Yan Sin, Abraham Tak Ka Man, Wai-Lun Law, Lui Ng

**Affiliations:** Department of Surgery, School of Clinical Medicine, Li Ka Shing Faculty of Medicine, The University of Hong Kong, Hong Kong SAR, China

**Keywords:** colorectal cancer, drug repurposing, antihypertensive drugs, hydrochlorothiazide, basophil, prognosis

## Abstract

**Background:**

Anti-hypertensive drugs have been reported to demonstrate anti-inflammatory and anti-angiogenic effects. This study aims to investigate the association between anti-hypertensive drugs and the prognosis of colorectal cancer (CRC) patients.

**Methods:**

Clinical data of 1134 CRC patients with hypertensions and the prescription of anti-hypertensive drugs who had undergone curative surgery in our hospital between 2005 and 2015 were retrieved. Their survival data and immune cell population in circulatory blood were compared among different types of anti-hypertensive drugs and overall CRC patients.

**Results:**

The 5-year overall survival for the antihypertensives-treated patients (65.2%) was higher than the CRC patients in Hong Kong (58.2%). Hydrochlorothiazide (HCTZ) group showed the best prognosis (79.1%) among different antihypertensive drug, particularly for advance stage or elderly patients, which are poor prognostic factors for overall CRC patients, demonstrated an obviously improved prognosis upon HCTZ treatment. Moreover, our data showed the recurrence rate was significantly lower for HCTZ group (18.3%) compared to non-HCTZ group (26.8%) and the reported rate (31%) of CRC patients in Hong Kong. Finally, patients with a lower pre-operative basophil level showed better overall and disease-free survival following HCTZ treatment.

**Conclusion:**

This study demonstrated the association of HCTZ treatment with a better prognosis of CRC patients.

## 1 Introduction

According to World Health Organization, colorectal cancer (CRC) is the third most common cancer worldwide, accounting for approximately 10% of all cancer cases and is the second leading cause of cancer-related deaths globally (Marcellinaro et al., 2023). The survival rates and quality of life for CRC patients are significantly low due to factors such as high mortality rates, late-stage diagnoses, metastasis to distant locations, and the limited effectiveness and adverse effects of standard treatments. Typically, CRC is treated using a single method or a combination of methods, including surgery, chemotherapy, and radiation therapy. While there has been some recent evidence of improved overall survival and disease-free survival in CRC patients, the 5-year survival rates remain below 65% in developed countries and below 50% in developing countries (Allemani et al., 2015). Furthermore, the 5-year relative survival rate is even poorer for CRC patients with distant metastases, ranging from 14% to 15% (Siegel et al., 2020). The bleak prognosis, especially for advanced-stage patients, underscores the urgent need for more effective treatments. In recent years, significant progress has been made in achieving long-term responses using immune checkpoint inhibitors (ICIs) for solid tumors like melanoma, non-small-cell lung cancer (NSCLC), and renal cell cancer (Webb et al., 2018). However, the majority of CRCs and CRC metastases do not exhibit deficient MisMatch Repair (dMMR), making most CRC patients ineligible for immunotherapy (Kamal et al., 2020). Therefore, there is an urgent need to identify better therapeutic targets for CRC patients. However, the process of developing new drugs involves lengthy preclinical and clinical trials before they are approved for use in CRC patients. As a result, drug repurposing has emerged as an attractive approach, utilizing already approved drugs to improve patient prognosis (Xia et al., 2024).

Anti-hypertensive drugs have been reported to demonstrate anti-inflammatory and anti-angiogenic effects (Nemati et al., 2011; Ferroni et al., 2012), whereas both inflammation and angiogenesis are closely related to development and progression of cancers (Aguilar-Cazares et al., 2019). Therefore we are interested to investigate the potential impacts of anti-hypertensive drugs on the prognosis of CRC patients. In fact, hypertension is one of the risk factors for the development of CRC, and many CRC patients have comorbidity of hypertension and are taking anti-hypertensive drugs (Kaneko et al., 2021). Hence there is a large patient population available for evaluating the potential therapeutic effect of different anti-hypertensive drugs. An American research group reported in June 2021 that anti-hypertensive drugs may improve survival in CRC (Balkrishnan et al., 2021), which is in line with our hypothesis. Their study used the Surveillance, Epidemiology, and End-Results (SEER) Medicare database to review outcomes of 13,982 patients aged 65 and older diagnosed with stage I-III colorectal cancer between 2007 and 2012, showing that patients treated with Angiotensin Converting Enzyme (ACE) inhibitors, beta blockers and thiazide diuretics were all associated with decreased cancer-specific mortality (Balkrishnan et al., 2021). The authors suggested that antihypertensive drugs could improve prognosis of CRC patients through functionally interacting with the tumor vasculature and microenvironment (Balkrishnan et al., 2021); however more clinical and experimental evidence is needed to support their repurposing for treating CRC patients.

**FIGURE 1:**
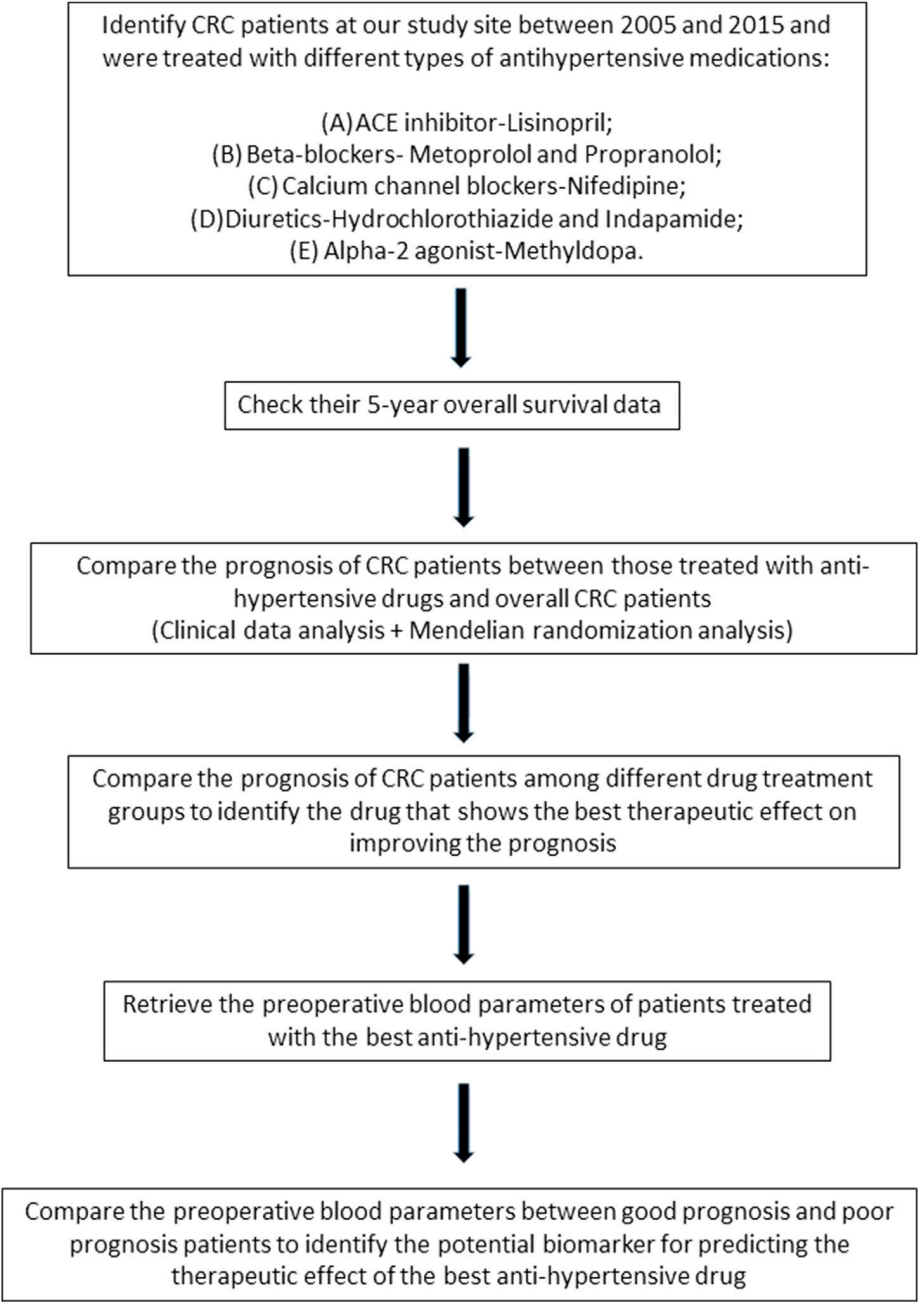
The flowchart of this study.

The objective of this study is to examine the therapeutic impact of various anti-hypertensive medications on enhancing the prognosis of CRC patients. The flowchart of this study is shown in Figure 1.

## 2 Materials and methods

### 2.1 Subjects

CRC patients who underwent curative surgery and had a history of prescription of anti-hypertensive drugsat Queen Mary Hospital (Hong Kong) during 2005–2015 were included in the study. These patients were treated with different types of antihypertensive medications. For this study, we selected 7 common drugs from 5 different classes, including (A) ACE inhibitor-Captopril, (B) Beta-blockers- Metoprolol and Propranolol; (C) Calcium channel blockers- nifedipine; (D) Diuretics-Hydrochlorthiazide and Indapamide; (E) Alpha-2 agonist-Methyldopa. Patients who were not followed up for at least 5 years were excluded, except for those who exhibited signs of recurrence or death during the follow-up period. The data collection protocol has been approved (UW 21-114) by the Institutional Review Board (IRB) of the University of Hong Kong.

### 2.2 Data retrieval from Hong Kong clinical data analysis and reporting system (CDARS)

A population-based retrospective cohort was compiled from the Hong Kong Hospital Authority (HA) administrative database in the Hong Kong adult diabetes population from 1 January 2006 to 31 December 2015. CDARS has been extensively utilized for conducting high-quality large population-based studies (Ng et al., 2021). The database encompasses comprehensive demographic and clinical characteristics of CRC patient (ICD-9-CM Diagnosis Code: 153.0–153.9; 154.0–154.3; 154.8), including the year of diagnosis, age, sex, race/ethnicity, tumor location, tumor size, tumor stage, receipt of cancer treatment (such as surgery, chemotherapy, and radiation therapy); patient’s immune cell profile including lymphocyte count, neutrophil count, neutrophil-to-lymphocyte ratio (NLR), platelet count, platelet-to-lymphocyte ratio (PLR), monocyte count, lymphocyte-to-monocyte ratio (LMR), albumin level and prognostic nutritional index (PNI); HA drug prescription database containing information, including the date of drug dispensing, dosage unit, and quantity of drug dispensed, which was recorded. The baseline date for eligible patients was defined as the date of curative resection of primary CRC. Patients were monitored from the baseline date until the incidence of the event outcome, death from any cause, and censored at the last healthcare service utilization date, whichever came first.

### 2.3 Statistical analysis

Univariate analysis of the association between recurrence or no recurrence and the treatment received was performed using Fisher’s exact test. Survival curves were generated using the Kaplan–Meier test and compared by the log-rank test. The difference in immune cell profile between treatment and control group was compared by t-test, paired-test or Mann–Whitney U test. The criterion for statistical significance was a *p* < 0.05. All statistical analyses are conducted using SigmaPlot version 10.0 (Systat Software Inc., San Jose, CA, United States) and SPSS version 10.0 (SPSS Inc., Chicago, IL, United States).

### 2.4 Mendelian Randomization

Mendelian Randomization (MR) is a causal inference method that utilizes germline genetic variants (specifically single nucleotide polymorphisms (SNPs)) as genetic instruments to estimate and test the causal effect of an exposure variable on an outcome (Burgess and Thompson, 2017). In this study, the R package TwoSampleMR was utilized to implement various MR methods for analyzing the association between phenotypes and survival, as described in a previous study (Xin et al., 2023). The methods employed included inverse variance weighted (IVW), weighted median, penalized weighted median, and MR Egger methods. Additionally, a heterogeneity test was conducted to evaluate whether a genetic variant’s effect on the outcome was proportional to its effect on the exposure, and the MR-Egger intercept test was performed to assess the presence of horizontal pleiotropy (Burgess and Thompson, 2017). Suggestive evidence of an association between phenotypes and cancer survival was identified when specific criteria were met, including a P-value ≤0.05 for IVW analysis, a P-value >0.05 for the Egger intercept, and a P-value >0.05 for heterogeneity. A two-sample MR analysis was carried out using the selected SNPs to identify potential therapeutic targets for tumors. In this particular investigation, antihypertensive drug usage (ebi-a-GCST90018984 - Medication use (antihypertensives) obtained from MRanalysis (https://mranalysis.cn/index.html)) was considered as the exposure factor, while cancer-specific survival (CSS) served as the outcome factor (Xin et al., 2023).

## 3 Results

### 3.1 Antihypertensive drugs improve prognosis of CRC patients

**FIGURE 2:**
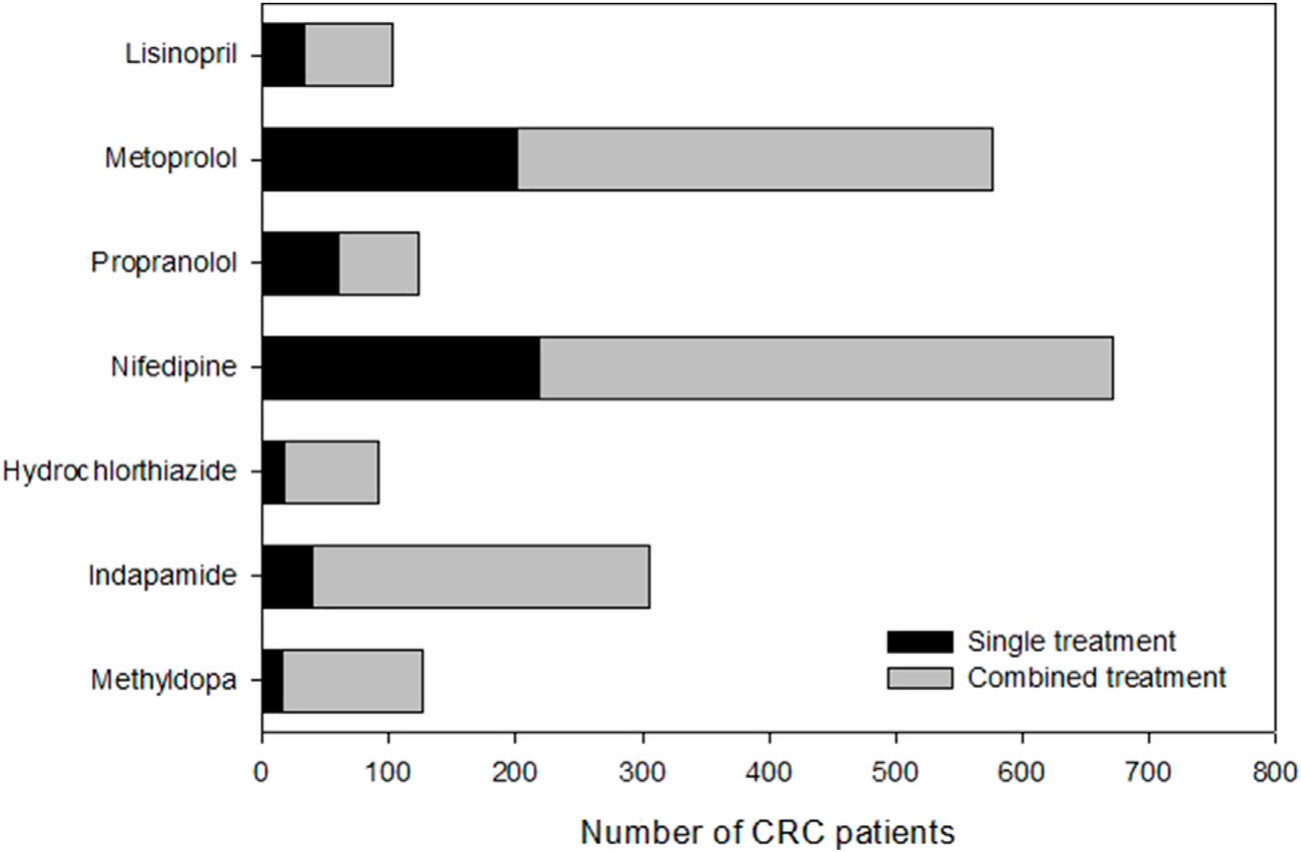
Number of CRC patients underwent surgery during 2005–2015 in Queen Mary Hospital and treated with antihypertensive drugs.

We compared the 5-year overall survival of CRC patients who underwent surgery at our study site (Queen Mary Hospital) between 2005 and 2015 and were treated with different types of antihypertensive medications. In this study, we selected 7 common drugs from 5 different classes, including (A) ACE inhibitor-Lisinopril; (B) Beta-blockers- Metoprolol and Propranolol; (C) Calcium channel blockers-Nifedipine; (D) Diuretics-Hydrochlorothiazide and Indapamide; (E) Alpha-2 agonist-Methyldopa. Table 1 shows the age, gender and tumor location information for the CRC patients in different antihypertensive drug groups. A total of 1,134 patients were found to be treated with these drugs either individually or in combination. The distribution of CRC patients in each anti-hypertensive drug group (single or combined drug) is shown in Figure 2.Our findings revealed that the 5-year overall survival for patients treated with antihypertensives-treated patients (65.2%) was higher than that of CRC patients in Hong Kong (58.2%) (Registry, 2019).

**TABLE 1:** Characteristics of eligible CRC patients in different anti-hypertensive drug groups.

	HCTZ	Indapamide	Nifedinpine	Metoprolol	Propranolol	Methyldopa	Lisinopril
Age							
>75	48	146	332	269	36	80	72
≤75	38	164	352	309	90	49	30
Gender							
M	40	155	385	345	46	65	69
F	46	155	289	233	80	64	33
Tumor location							
Colon	62	229	478	415	95	99	67
Rectum	24	71	196	163	31	30	35

### 3.2 MR analysis

**FIGURE 3:**
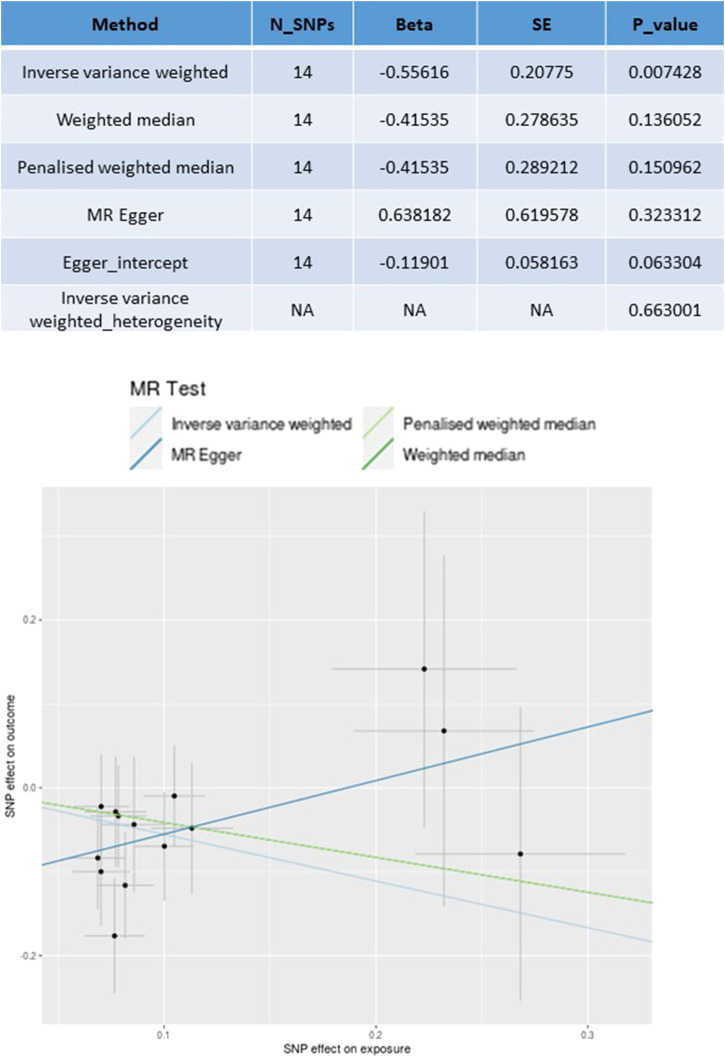
Mendelian randomization analyses for antihypertensive drugs use (exposure) on cancer-specific survival (CSS) outcome.

Among the 2,621 colorectal cancer (CRC) patients included in the MR analysis, 569 cancer-specific deaths were recorded (Xin et al., 2023). As shown in Figure 3, the analysis revealed a significant effect of antihypertensive drugs on CRC CSS (beta = −0.5561, P = 0.007) using IVW with 14 SNPs. No substantial directional pleiotropy was observed based on the MR-Egger test results (MR-Egger intercept 0.638, P > 0.05). Furthermore, no significant heterogeneity was detected when comparing the effect sizes of instrumental variables (IVs) on exposure to their effect sizes on CRC CSS survival (P_heterogeneity_ > 0.05). The MR estimates indicated that antihypertensive drug usage was associated with improved CSS in CRC, consistent with the findings of the clinical study.

### 3.3 CRC patients treated with hydrochlorothiazide (HCTZ) showed the best prognosis

**FIGURE 4:**
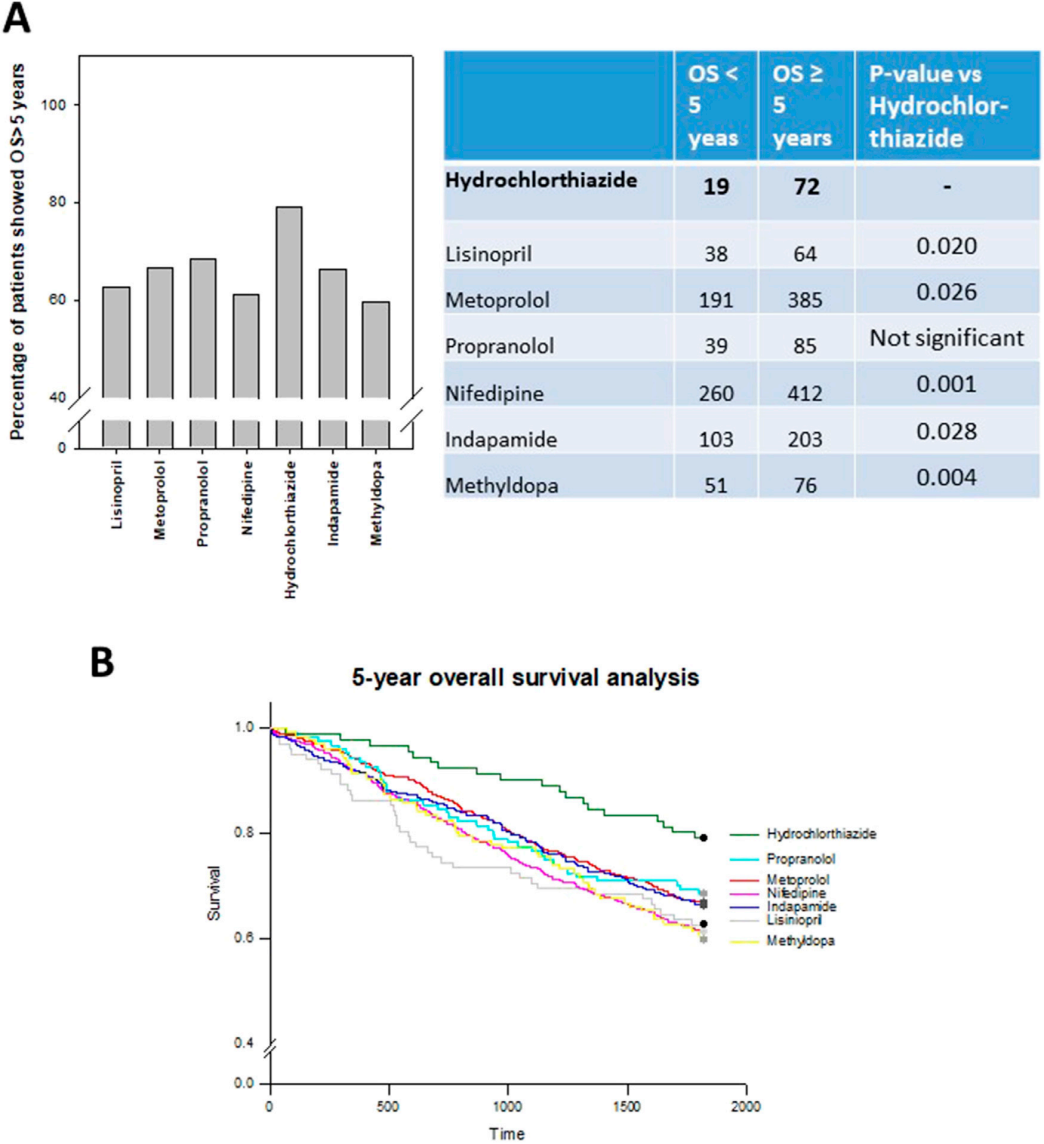
5-year overall survival (OS) analysis of different antihypertensive drugs treated CRC patients **(A)** Percentage of patients who can live more than 5 years post-surgery. Hydrochlorthiazide (HCTZ) group showed better prognosis when compared to other antihypertensive drugs, and the difference was significant for some of the comparison as shown in the table. **(B)** Log-rank analysis showing the overall survival of patients in different drug groups. Hydrochlorthiazide group was obviously better than the remaining drug groups.

Hydrochlorothiazide (HCTZ) group exhibited the best prognosis (79.1%) among the different antihypertensive drug groups, and the results were statistically significant when compared to Lisinopril, Metoprolol, nifedipine, Indapamide and Methyldopa (Figures 4A, B).

**FIGURE 5:**
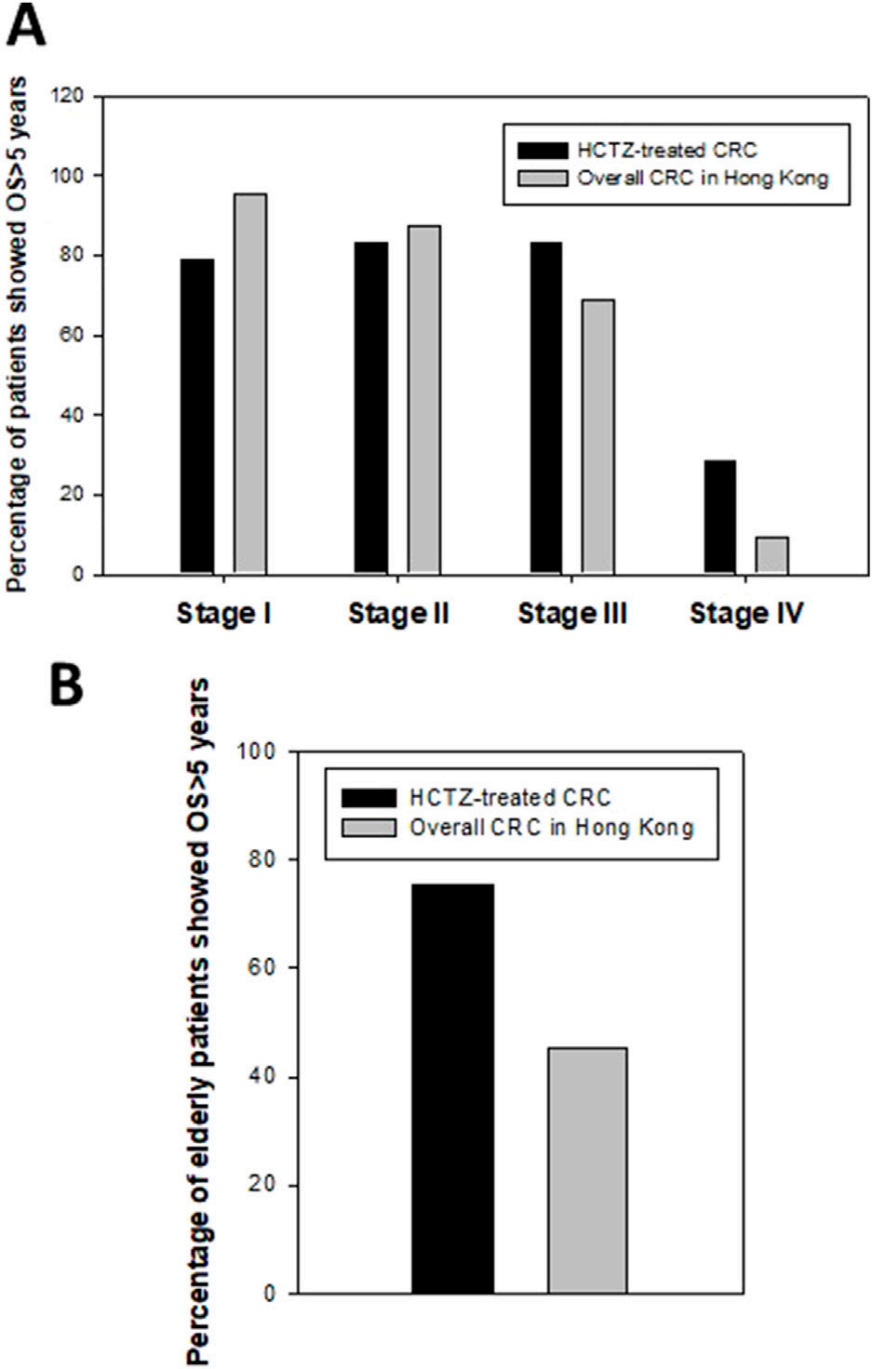
Effect of Hydrochlorthiazide (HCTZ) on prognosis of advance stage or elderly CRC patients **(A)** Stage-specific 5-year overall survival (OS) data of HCTZ-treated CRC patients and overall CRC patients in Hong Kong. The prognosis of advance stage group (Stage III and IV) in HCTZ group was better than the overall CRC patients in Hong Kong. **(B)** Comparison of 5-year overall survival (OS) of elderly patients between HCTZ-treated group and overall data in Hong Kong.

The 5-year overall survival rates for different stages of the HCTZ group were as follows: stage I: 15 out of 19, 78.9%; stage II: 25 out of 30, 83.3%; stage III: 25 out of 30, 83.3%; and stage IV: 2 out of 7, 28.6%, whereas the rates for stage III and IV were notably higher than those of overall CRC patients in Hong Kong (68.7% and 9.3%, respectively, as per the “Overview of Hong Kong Cancer Statistics of 2018” published by the Hospital authority) (Figure 5A). The relatively lower 5-year overall survival rates for stage I and II patients in the HCTZ group were likely due to the higher average age of patients in these groups (74.8 for stage I and 75.4 for stage II). In addition, the 5-year overall survival rate for elderly patients (age 75 or above) was the worst among different age groups for overall CRC patients in Hong Kong. Our data indicated that the rate for elderly patients in HCTZ group was 75.5%, which was much higher than the overall rate for elderly CRC patients (45.2%) (Figure 5B). These findings suggest that advanced stage or elderly CRC patients, which are poor prognostic factors for overall CRC patients in Hong Kong, showed a noably improved prognosis with HCTZ treatment.

**FIGURE 6:**
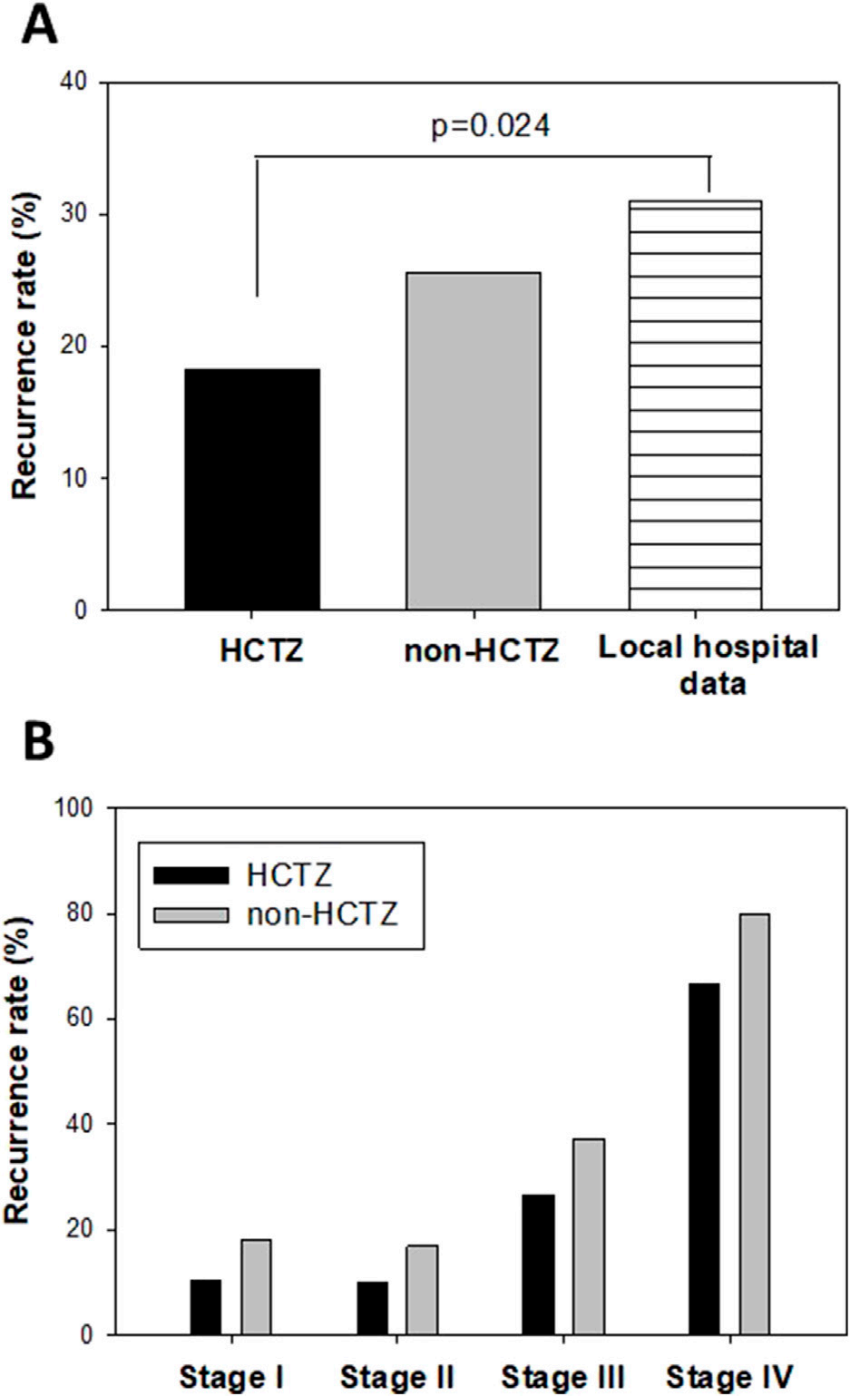
Effect of Hydrochlorthiazide (HCTZ) on disease recurrence of CRC patients who performed curative surgery **(A)** Comparison of overall recurrence rate among HCTZ-treated patients, non-HCTZ antihypertensive drug treated patients and data reported from a local hospital which included 650 CRC patients who underwent curative-intent resection between 2000 and 2006 (KC Cheng et al., 2008). **(B)** Comparison of stage-specific recurrence rate among HCTZ-treated and non-HCTZ antihypertensive drug treated patients in this study.

Furthermore, we investigated the association between HCTZ treatment and the recurrence of CRC patients who underwent curative surgery. Among our study cohort, we identified 82 CRC patients treated with HCTZ who underwent curative surgery, and we included 190 age-, gender- and tumor stage-matched non-HCTZ antihypertensive drug-treated patients for comparison. Table 2 shows the age, gender and tumor stage information for the CRC patients in the HCTZ and non-HCTZ groups. Our data revealed that 15 out of the 82 patients (18.3%) in the HCTZ group developed recurrent disease, which was lower than the rate in non-HCTZ patients (51 out of 190 patients (26.8%)). This recurrence rate was also significantly lower (p = 0.024) than the data reported from another local hospital (Cheng et al., 2008), which mentioned that 31% of 650 CRC patients who underwent curative-intent resection between 2000 and 2006 developed recurrent disease (Figure 6A).

**TABLE 2:** Characteristics of eligible CRC patients in the HCTZ and non-HCTZ groups.

	HCTZ	Non-HCTZ
Age		
>75	44	99
≤75	38	91
Gender		
M	39	87
F	43	103
Tumor stage		
I/II	49	110
III/IV	33	80

We also compared the recurrence rate of CRC patients at each stage between the HCTZ and non-HCTZ groups. As shown in Figure 6B, the recurrence rates in HCTZ at different stages of CRC (stage I: 2 out of 19, 10.5%; stage II: 3 out of 30, 10%; stage III: 8 out of 30, 26.7%; stage IV: 2 out of 3, 66.7%) were lower for all stages compared to the non-HCTZ group (stage I: 6 out of 33, 18.2%; stage II: 13 out of 77, 16.9%; stage III: 28 out of 75, 37.3%; stage IV: 4 out of 5, 80.0%).

### 3.4 Preoperative blood basophil level is associated with prognosis of HCTZ-treated CRC patients

**FIGURE 7:**
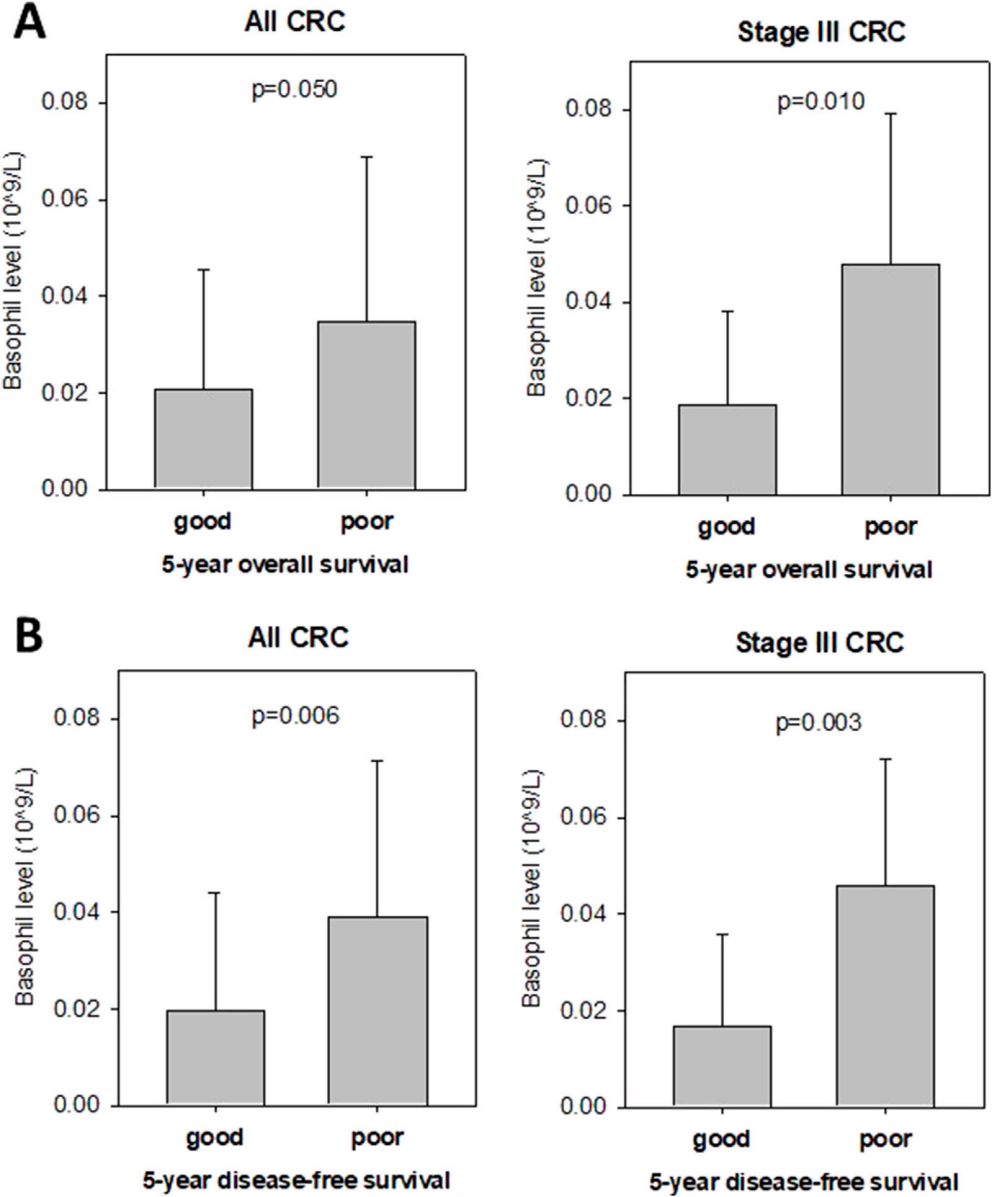
Pre-operative basophil level is associated with prognosis of Hydrochlorthiazide (HCTZ) –treated CRC patients **(A)** Level of pre-operative basophil in good or poor prognosis overall CRC patients (left) and stage III CRC patients (right) based on their 5-year overall survival status. It was shown that low basophil level was associated with good prognosis. **(B)** Level of pre-operative basophil in good or poor prognosis overall CRC patients (left) and stage III CRC patients (right) based on their 5-year disease-free survival status after curative surgery. It was shown that low basophil level was associated with good prognosis.

Anti-hypertensive drugs, including HCTZ, have been reported to exhibit anti-inflammatory and anti-angiogenic effects (Nemati et al., 2011; Ferroni et al., 2012). We investigated whether pre-operative blood parameters could serve as potential biomarkers for predicting CRC patients with a good prognosis following HCTZ treatment. Among all circulatory blood parameters, we found that the pre-operative basophil level was significantly associated with overall survival and disease-free survival (Figure 6). Figure 7A shows that patients with a good prognosis in terms of overall survival had a significantly lower pre-operative basophil level (0.0209 × 10^9^/L) compared to the poor prognosis group (0.0347 × 10^9^/L, p = 0.05). The difference was particularly significant for stage III CRC, whereas the basophil level in the good prognosis group (0.0188 × 10^9^/L) was significantly lower than that in the poor prognosis group (0.0480, p = 0.01). For disease-free survival analysis (Figure 7B), patients with a good prognosis also exhibited a significantly lower pre-operative basophil level (0.0197 × 10^9^/L) compared to the poor prognosis group (0.0389 × 10^9^/L, p = 0.0099). Once again the difference was particularly significant for stage III CRC (0.0168 × 10^9^/L vs. 0.0457 × 10^9^/L, p = 0.0034). These findings, if validated using a larger patient population, could provide a potential biomarker for identifying CRC patients who may have a better prognosis following HCTZ treatment.

## 4 Discussion

Colorectal cancer (CRC) is a prevalent malignant cancer worldwide, with increasing incidence and mortality rates (Marcellinaro et al., 2023). While targeted drugs and therapeutic regimens have been developed and proven effective in treating CRC, there is still a significant number of patients who lack effective targeted drugs or develop drug resistance during treatment (Kumar et al., 2023). Therefore, there is an urgent need to develop new therapeutic agents. Drug repurposing for cancer therapy is a promising strategy for discovering new drugs. Compared to developing new drugs from scratch, drug repurposing significantly reduces the time, investment, and improves the success rates of preclinical drug discovery (Carlos-Escalante et al., 2021). Antihypertensive drugs, widely prescribed and available at an affordable price with a well-known safety profile, are particularly interesting for repurposing (Carlos-Escalante et al., 2021). Additionally, the large number of cancer patients undergoing antihypertensive drug treatment allows for the analysis of their potential impact on cancer treatment.

In June 2021, an American research group reported that antihypertensive drugs may improve survival in CRC (Balkrishnan et al., 2021). Their study, using the Surveillance, Epidemiology, and End-Results (SEER) Medicare database, reviewed outcomes of 13,982 patients aged 65 and older diagnosed with stage I-III colorectal cancer between 2007 and 2012. The study found that patients treated with Angiotensin Converting Enzyme (ACE) inhibitors, beta blockers, and thiazide diuretics had decreased cancer-specific mortality (Balkrishnan et al., 2021). Specifically, anti-hypertensive drugs were associated with decreased cancer-specific mortality, with ACE inhibitors, beta-blockers and thiazide diuretics showing positive results (Balkrishnan et al., 2021). The authors further suggested that hypoxia and irregular tumor vascularization can contribute to treatment failure by promoting metastasis, complicating surgery, and limiting the efficacy of known cancer therapies. Strategies that normalize tumor vasculature function and hypoxia through anti-hypertensive drugs to improve the tumor microenvironment could be effective in optimizing cancer patient management (Balkrishnan et al., 2021). However, more clinical and experimental evidence is required to support the repurposing of antihypertensive drugs for treating CRC patients. So far, β-blockers, ACE inhibitors, and ARBs have been the most commonly evaluated antihypertensive agents in the context of cancer, while thiazide and thiazide-like diuretics have been less studied (Carlos-Escalante et al., 2021). CRC is a heterogeneous disease, and this diversity can have important implications for CRC prognosis and clinical management (Kocarnik et al., 2015). Therefore, it is uncertain whether the same effect of antihypertensive drugs observed in CRC patients in the United States will be seen in the Asian population. This study offers additional clinical evidence that antihypertensive drugs, particularly HCTZ, could enhance the prognosis of CRC patients. Additionally, the study examined the effects of commonly used specific antihypertensive drugs. It was found that within the diuretic drug group, the HCTZ group demonstrated a better prognosis (79.1%) compared to the indapamide group (66.3%).

This study analyzed CRC patients with concurrent hypertension in a single hospital in Hong Kong and compared the prognosis among CRC patients treated with various types of antihypertensive drugs. Our findings showed that the overall 5-year survival rate for antihypertensive-treated patients (64.4%) was higher than that reported for CRC patients in Hong Kong (58.2%). Specifically, the hydrochlorothiazide (HCTZ) group had the best 5-year overall survival rate (79.1%) among different antihypertensive drug groups. Additionally, the recurrence rate for CRC patients in the HCTZ group was lower than that of non-HCTZ patients, while both groups had lower recurrence rates than those reported from another local hospital for overall CRC patients (Cheng et al., 2008). These findings suggest that antihypertensive drugs, particularly HCTZ, have the potential to improve the prognosis of post-operative CRC patients. In the future, we plan to validate these findings further in a larger cohort of patients in multiple hospitals in Hong Kong and nearby regions. Additionally, we will investigate the best drug in combination with HCTZ for CRC patients in terms of both disease-free and overall survival. It is also necessary to investigate the functional effect of HCTZ *in vitro*, *in vivo*, or *ex vivo*.

It is important to note that HCTZ has known photosensitizing properties (Haisma et al., 2023), and some studies have reported an association between the cumulative use of HCTZ and cancer development (Rahamimov et al., 2024; Shin et al., 2019; VanWormer et al., 2022). The risk of any skin cancer, keratinocyte carcinoma, basal cell carcinoma, and squamous cell carcinoma was not significantly increased in general HCTZ users (Haisma et al., 2023). However, a clear association was observed between high cumulative HCTZ use (≥5,000 defined daily dose; ≥125,000 mg) and the risk of any skin cancer, keratinocyte carcinoma, basal cell carcinoma, and squamous cell carcinoma (Haisma et al., 2023; Park et al., 2020). Nevertheless, some studies have questioned this correlation, and whether HCTZ increases the risk of skin cancer remains controversial (Park et al., 2020). A nationwide case-control study reported that the use of HCTZ was associated with a substantially increased risk of lip cancer, and the risk increased with the increasing cumulative amount, duration, and intensity of HCTZ use (Pottegard et al., 2017). More than 100,000 mg of HCTZ, corresponding to more than 10 years of cumulative use, was associated with a seven-fold increased risk for squamous cell carcinoma lip cancer (Pedersen et al., 2018). On the other hand, a recent study investigated the association between HCTZ usage and the risk of melanoma (Park et al., 2020). The risk of melanoma was significantly lower in HCTZ-users compared to non-HCTZ users. High cumulative doses (≥50,000 mg) of HCTZ were associated with a decreased risk of both non-melanoma skin cancer and melanoma (Park et al., 2020).

This study possesses several notable strengths. Firstly, we conducted a thorough examination of the clinical effects of various common antihypertensive drugs across different categories on CRC patients. This analysis was based on comprehensive follow-up data obtained from CRC patients treated at our hospital, providing valuable insights into the impact of these medications on CRC outcomes. Secondly, a significant strength of this study lies in our identification of a blood biomarker capable of distinguishing CRC patients who are likely to experience improved prognoses when treated with HCTZ. This discovery not only contributes to a more personalized approach to treatment but also underscores the potential for utilizing biomarkers to tailor therapeutic strategies for individual patients. However, there are also limitations to this study. Firstly, it is a single-center study, and multicenter data is necessary to increase the sample size for each type of antihypertensive drug and validate our findings. Secondly, the functional therapeutic effects of HCTZ on CRC have not been explored, which could offer insight into how it enhances the prognosis of CRC patients and potentially improve its clinical applicability. For example, angiogenesis plays a pivotal role in tumor growth, invasion, and metastasis (Liu et al., 2023). Research indicates that HCTZ inhibits RhoA and Rho-associated protein kinase (ROCK) transcription and activation in mouse models (Mondaca-Ruff et al., 2021; Araos et al., 2016), and the RhoA/ROCK pathway is known to be crucial in angiogenesis (Chen et al., 2014). Furthermore, studies have shown that ROCK inhibitors reduce VEGF-induced angiogenesis and tumor cell growth, leading us to hypothesize that HCTZ exerts an anti-angiogenic effect by inhibiting the ROCK pathway.

## 5 Conclusion

This study demonstrated the association between HCTZ treatment and a better prognosis for CRC patients. Post-operative patients treated with HCTZ exhibited significantly enhanced 5-year overall survival and a lower recurrence rate compared to those treated with non-HCTZ antihypertensive drugs or the overall CRC patient population. Furthermore, our results demonstrated for the first time that the pre-operative basophil level was significantly associated with overall survival and disease-free survival following HCTZ treatment, suggesting that the pre-operative basophil level could be a potential biomarker for identifying patients who are likely to respond better to HCTZ treatment.

## Data Availability

The raw data supporting the conclusions of this article will be made available by the authors, without undue reservation.
